# Effectiveness of visual inspection, practices observation and aerobic colony count to monitor hospital cleanliness

**DOI:** 10.1017/ash.2025.79

**Published:** 2025-09-03

**Authors:** Ying-Chun Chen, Pei-Yi Lin, Hui-Mei Huang, Zhi-Yuan Shi, Chun-Hsi Tai

**Affiliations:** 1 Infection Control Center, Taichung Veterans General Hospital, Taichung, Taiwan; 2 Nursing Department, Taichung Veterans General Hospital, Taichung, Taiwan

## Abstract

**Background:** Enhancing environmental hygiene resulted in a reduction of multidrug-resistant microorganisms colonization and healthcare-associated infections. There has been less studies to compare the effects of practice observation with other methods. This study aimed to compare correlations between visual inspection, practice observation and aerobic colony count (ACC) and verify the effectiveness. **Methods:** A prospective study was conducted in a medical intensive care unit from May 2021 to November 2022. High-touch surfaces were assessed by visual inspection (clean or not clean) and practice observation (compliant or not compliant) to compare the correlations by using ACC with the cut-off point of 2.5 CFU/cm^2^ as a golden standard. **Results:** Among 569 samples, the pass rate by ACC was 90.5%, the clean rate by visual inspection was 73.3%, and the compliant rate by practice observation was 47.1%. The concordance was 245 surfaces (43.1%) of the three methods. There was no correlation between visual inspection and ACC (p<0.001, φ=0.184). The correlations were weak positive between visual inspection and practice observation and between practice observation and ACC (p<0.001, φ=0.212, 0.233). The median aerobic colony count of “compliant” group (0.00 CFU/cm^2^) was significantly lower than “not compliant” (0.40 CFU/cm^2^) (p<0.001). The median aerobic colony count of “clean” groups (0.08 CFU/cm^2^) was also significantly lower than “not clean” groups (0.20 CFU/cm^2^) (p<0.001). **Conclusion:** Practice observation is more reliable than visual inspection. Therefore, visual inspection *can be used for low risk area to maintain* visibly clean. In high *risk area, an integrated program is critical to* combine practice observation w*ith other methods to monitor cleanliness.*

